# A biomechanical comparison between cement packing combined with extra fixation and three-dimensional printed strut-type prosthetic reconstruction for giant cell tumor of bone in distal femur

**DOI:** 10.1186/s13018-022-03039-y

**Published:** 2022-03-09

**Authors:** Xin Hu, Minxun Lu, Yuqi Zhang, Yitian Wang, Li Min, Chongqi Tu

**Affiliations:** Department of Orthopedics, Orthopedic Research Institute, West China Hospital, Sichuan University, No. 37 Guoxuexiang, Chengdu, 610041 Sichuan People’s Republic of China

**Keywords:** Giant cell tumor, Distal femur, 3D-printed prosthesis, Bone cement, Finite element analysis

## Abstract

**Background:**

The most common reconstruction method for bone defects caused by giant cell tumor of bone (GCTB) is cement packing combined with subchondral bone grafting and extra fixation. However, this method has several limitations involving bone cement and bone graft, which may lead to poor prognosis and joint function. A titanium-based 3D-printed strut-type prosthesis, featured with excellent biocompatibility and osseointegration ability, was developed for this bone defect in our institution. The goal of this study is to comparatively analyze the biomechanical performance of reconstruction methods aimed at the identification of better operative strategy.

**Methods:**

Four different 3D finite element models were created. Model #1: Normal femur; Model #2: Femur with tumorous cavity bone defects in the distal femur; Model #3: Cavity bone defects reconstructed by cement packing combined with subchondral bone grafting and extra fixation; Model #4: Cavity bone defects reconstructed by 3D-printed strut-type prosthesis combined with subchondral bone grafting. The femoral muscle multiple forces were applied to analyze the mechanical difference among these models by finite element analysis.

**Results:**

Optimal stress and displacement distribution were observed in the normal femur. Both reconstruction methods could provide good initial stability and mechanical support. Stress distributed unevenly on the femur repaired by cement packing combined with subchondral bone grafting and extra fixation, and obvious stress concentration was found around the articular surface of this femur. However, the femur repaired by 3D-printed strut-type prosthetic reconstruction showed better performance both in displacement and stress distribution, particularly in terms of the protection of articular surface and subchondral bone.

**Conclusions:**

3D-printed strut-type prosthesis is outstanding in precise shape matching and better osseointegration. Compared to cement packing and extra fixation, it can provide the almost same support and fixation stiffness, but better biomechanical performance and protection of subchondral bone and articular cartilage. Therefore, 3D-printed strut-type prosthetic reconstruction combined with subchondral bone grafting may be evaluated as an alternative for the treatment of GCTBs in distal femur.

## Introduction

In 1987, Campanacci et al. radiographically classified giant cell tumor of bone (GCTB), a benign but aggressive primary bone tumor, into three grades according to their level of bone destruction [1, 2]. For grades I or II GCTBs in distal femur, extended intralesional curettage is the standard therapeutic method [1, 3], and then the repairment of cavity bone defects seems highly demanded to improve postoperative outcomes.

The most popular reconstruction method is cement packing combined with subchondral bone grafting and extra fixation. Bone cement can perfectly match the osseous voids and provide sufficient mechanical strength, and it has a tumoricidal ability by thermal polymerization, which can adversely damage articular cartilage as well [4, 5]. The subchondral bone grafting (≥ 1 cm) not only can increase the thickness of subchondral bone if bone union occurs, but also acts as an allograft buffer to prevent thermal damage of the chondrocytes. Although early complications can be prevented, the long-term effectiveness is unknown. In fact, some patients still developed mechanical failure during long-term follow-up [6]. The main disadvantages include two aspects. Firstly, bone cement is too poorly osteoinductive and osteoconductive to achieve biological reconstruction and osseointegration of graft-cement interface. The different elastic modulus between the bone cement and the graft bone may result in absorbing of the surrounding bone [7]. Secondly, the source of autogenous bone is limited, and those irregular autogenous bone can hardly precisely match the bone defects contour, especially for the patellofemoral and tibiofemoral joints simultaneously. Besides, the rate of recurrence after curettage of GCTB can be reduced to an acceptable level by using several adjuvant methods including the use of phenol, ethanol, liquid nitrogen, and high-speed burr [8–11]. Given the disadvantages of using bone cement here, the tumoricidal ability is not the only justification for choosing it as a reconstruction material.

Porous design of titanium implants is receiving increasingly attention in the field of orthopaedics. These customized porous prostheses have several advantages such as excellent osseointegration ability, matchable shape, and low requirement for the amount of bone graft. Our previous studies reported good results after 3D-printed porous implant reconstruction with excellent osteointegration in eight patients with GCTB in proximal tibia at a middle-term follow-up [12, 13]. In the light of experience, a novel 3D-printed strut-type prosthesis had been designed to repair grade I or II GCTBs in distal femur. Furthermore, theoretical investigation using finite element analysis (FEA), a non-invasive method which has been reported as a promising method to investigate the stability and functionality of bone constructs [14, 15], are important for understanding the newly designed implant. Herein two surgical approaches for the repairment of cavity bone defects in distal femur, 3D-printed strut-type prosthetic reconstruction and cement packing combined with extra fixation, are compared both clinically and biomechanically in this study aimed at the identification of better operative strategy.

## Methods

### Clinical study

Patients with Campanacci I or II GCTB in distal femur, and patients who underwent extended curettage followed by bone defects reconstruction and subchondral bone grafting were included in the study. Patients with severe osteoporosis, patients with lower extremity deformities or abnormal muscle strength, and patients who had incomplete follow-up information were excluded. Between August 2019 and December 2020, 5 patients who underwent 3D-printed strut-type prosthetic reconstruction at our institute were selected for the present study, and another 5 patients who underwent cement packing combined plate-screws fixation and had similar tumor size to that of the 3D-printed group were selected as the control group.

There were 7 males and 3 females with a mean age of 35.3 ± 7.8 years (range 29–44 years). All patients underwent preoperative knee X-ray, femoral 3D-CT, knee MRI, and computed tomography (SPECT). The affected subchondral bone area proportion was evaluated before surgery according to the method described by Chen [16]. The basic patient information is summarized in Table 1.

**Table 1:** Basic information of GCTB patients

Patients	Sex	Age	The affected subchondral bone area proportion (%)	Campanacci grade	Reconstruction method	Follow-up (month)
1	M	44	27.2	II	Prosthesis	24
2	M	29	40.0	II	Prosthesis	27
3	M	29	24.1	II	Prosthesis	24
4	F	35	25.5	II	Prosthesis	24
5	F	33	18.2	I	Prosthesis	22
6	M	48	27.2	II	Cement pacing	26
7	M	29	26.1	II	Cement pacing	30
8	M	46	14.6	II	Cement pacing	22
9	F	33	46.7	II	Cement pacing	24
10	M	27	21.2	I	Cement pacing	24

All patients were regularly followed up for 24.7 ± 2.4 months (range 22–30 months). The follow-up contents included physical examination, clinical symptoms, and imaging examinations. The osteointegration of the bone/prosthesis interface was evaluated by Tomosynthesis Shimadzu Metal Artefact Reduction Technology (T-SMART). The functional outcome was assessed by the Musculoskeletal Tumor Society (MSTS) score.

### Biomechanical study

The CT-scanning data of femur used in this study were derived from a healthy middle-aged male volunteer and a similar-aged patient who had similar femoral anatomical features but a typical GCTB lesion in distal femur. (Our Ethical Committee authorized the study, and all people provided written informed consent to participate in this investigation).

#### The novel design of 3D-printed strut-type prosthesis

**Fig. 1:**
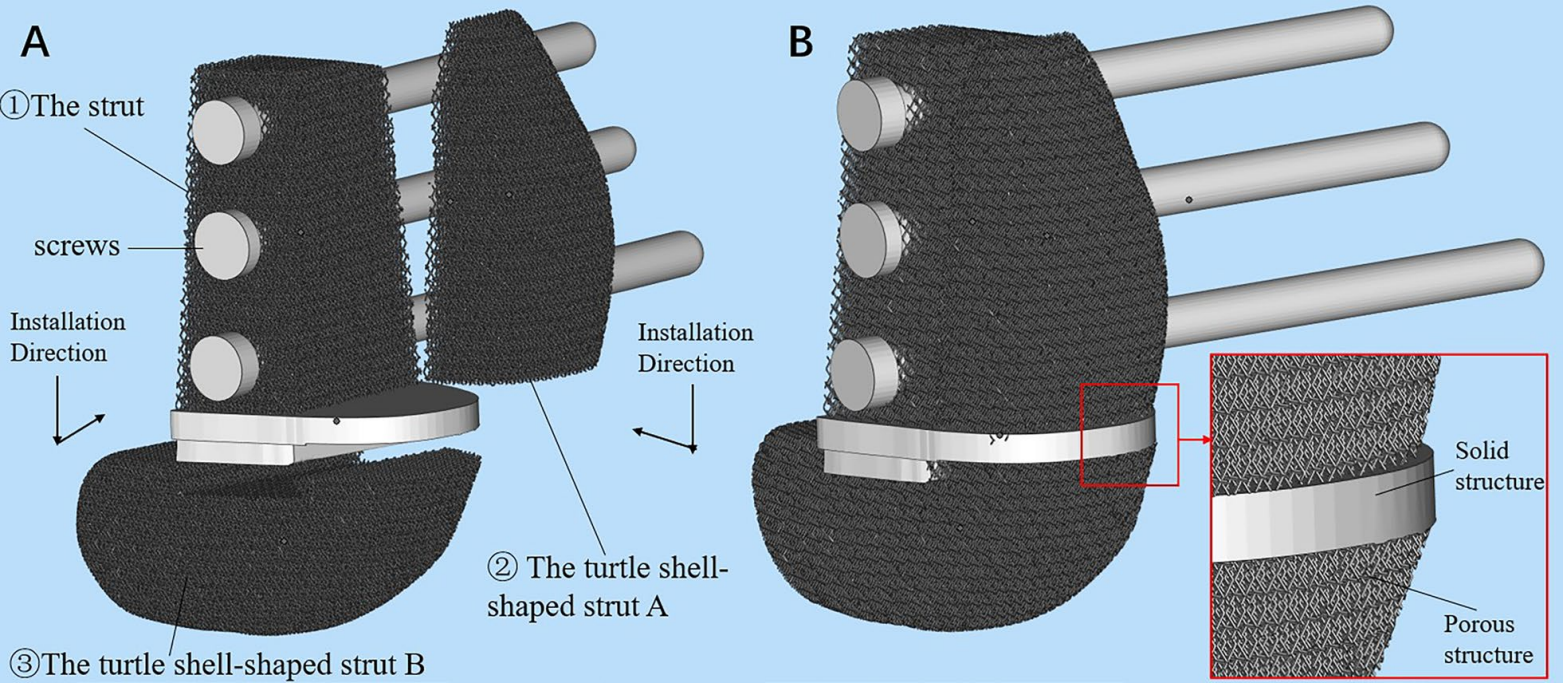
Diagram of the novel design of 3D-printed strut-type prosthesis: **A** the detached prosthesis, **B** the assembled prosthesis

The 3D-printed strut-type prosthesis was designed by our clinical team, using Solidworks 2016 (Dassault Systèmes SolidWorks Corporation, France). A modular system was applied to minimize the size of cortical windows and to make it convenient for assembling the components in the limited space. The modular system consisted of three components: ① The trapezoid-shaped strut was created to provide effective axial support, and three screws were fixed into the parallel holes through the strut in order to achieve transverse stability. ② The turtle shell-shaped strut A. ③ The turtle shell-shaped strut B was designed for maintaining the stability of patellofemoral and tibiofemoral joints and preventing the collapse of the articular surfaces, respectively. A specialized sideway-slider construction has been designed to establish a tight connection between the trapezoid-shaped strut and the turtle shell-shaped strut B. The slideway was built on the turtle shell-shaped strut B, and the corresponding slider was required to be located on the bottom of the trapezoid-shaped strut. Additionally, this prosthesis was made up of two different materials including the porous titanium (Ti6A14V) and the solid titanium (Ti6A14V). The porous titanium with scaffold structure (dark gray region in Fig. 1) can promote osseointegration of the bone–prosthesis interface, while the solid titanium (light gray region in Fig. 1) can provide effective mechanical strength.

#### Finite element models

##### Model #1: normal femur

**Fig. 2:**
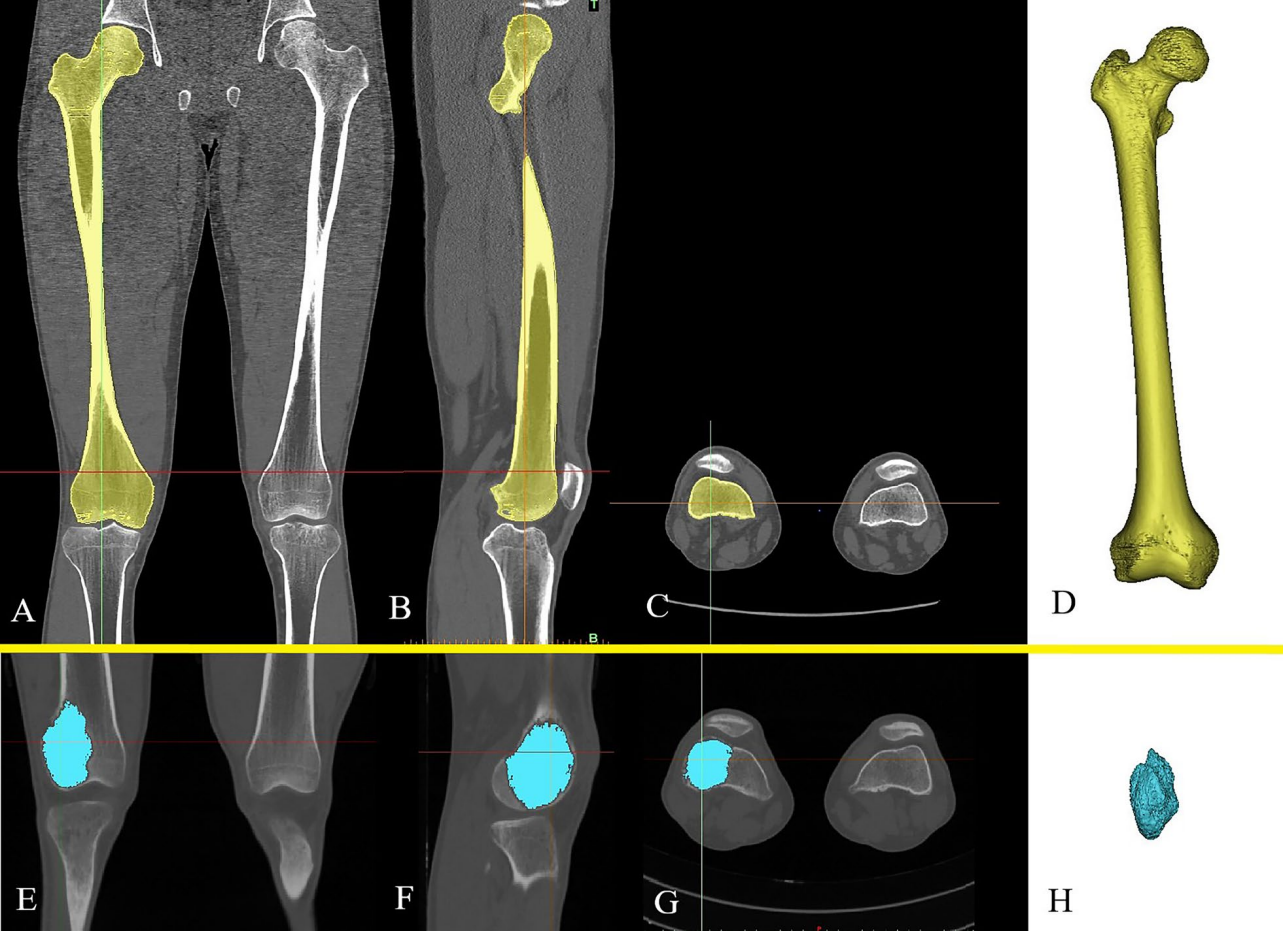
The reconstruction of the normal femur model and the tumor model: **A** Coronal, **B** sagittal, and **C** axial CT images of the normal femur. **D** The normal femur model. **E** Coronal, **F** sagittal, and **G** axial CT images of the femur with GCTB. **H** The tumor model

The CT-scanning data of the healthy volunteer were reconstructed three-dimensionally as the normal femur model using the software Mimics V17.0 (Materialise Corp. Belgium), as shown in Fig. 2A–D. Then, the initial model was further transformed into a solid model with None-Uniform Ration Basis Spine (NURBS) kyrtograph by the software Geomagic studio 2014 (3D Systems, Inc. USA).

##### Model #2: femur with tumorous cavity bone defects

**Fig. 3:**
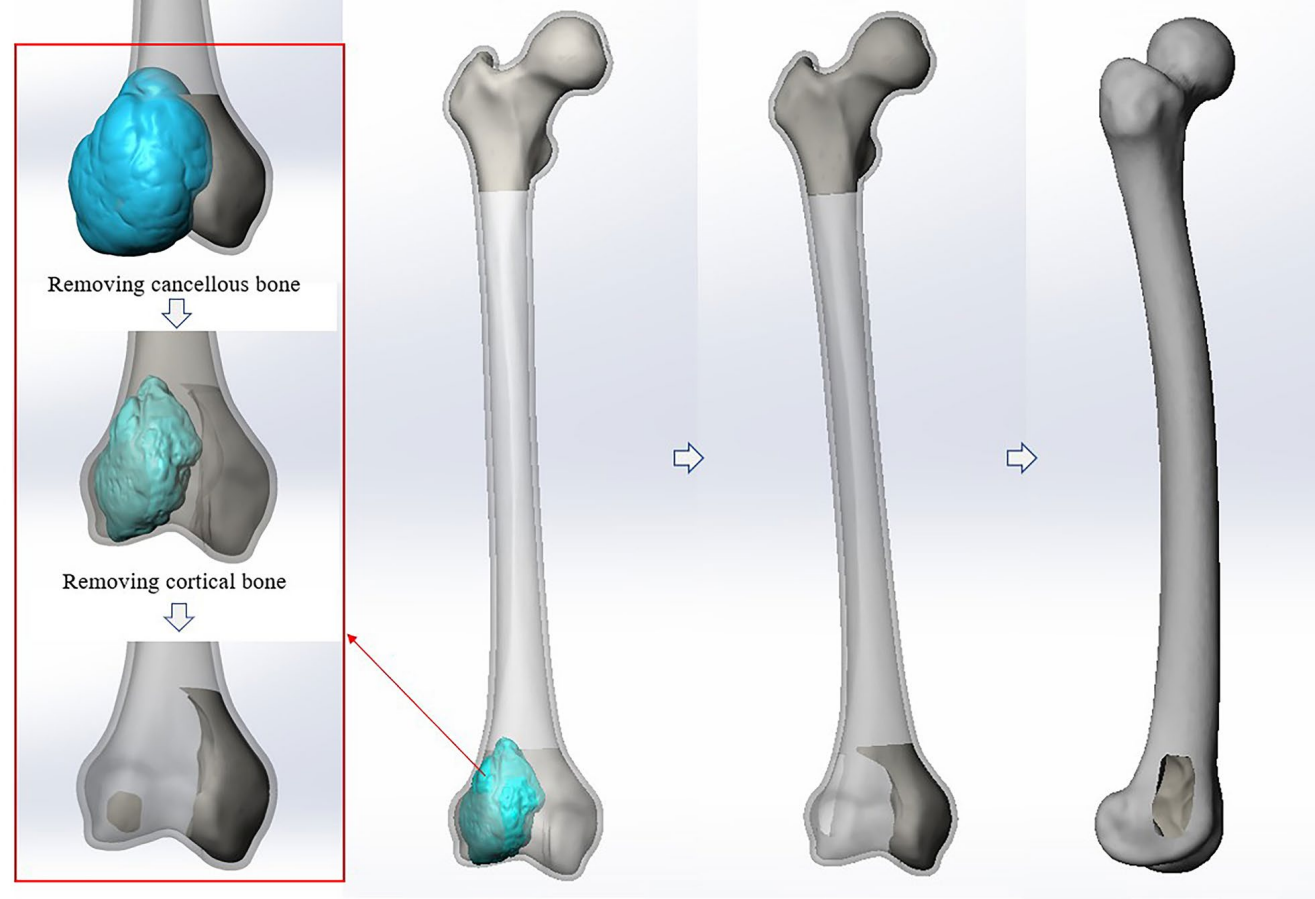
Diagram of the femur model with tumorous bone defects. The surgical procedures, creating cortical window and intralesional extended curettage, had been simulated by executing modules of Offsetting polygons and Boolean Operation in Solidworks 2016

3D CT-scanning data of GCTB patient were used to create tumor model (Fig. 2E–H), and then the tumor model and the normal femur model were assembled to simulate the tumorous bone defects in the distal femur with the same size (59.1 mm × 42.3 mm × 71.2 mm), shape (ellipsoid shape), and location (the distal lateral condyle) as those of the GCTB patient. The femur model with tumorous bone defects was subsequently imported into Solidworks 2016 for the purpose of simulating the surgical procedures, including creating cortical window and intralesional extended curettage. All surgical procedures were performed on the guidance of a senior surgeon (Li Min) from our clinic team (Fig. 3).

##### Model #3: cavity bone defects reconstructed by cement packing combined with subchondral bone grafting and extra fixation

**Fig. 4:**
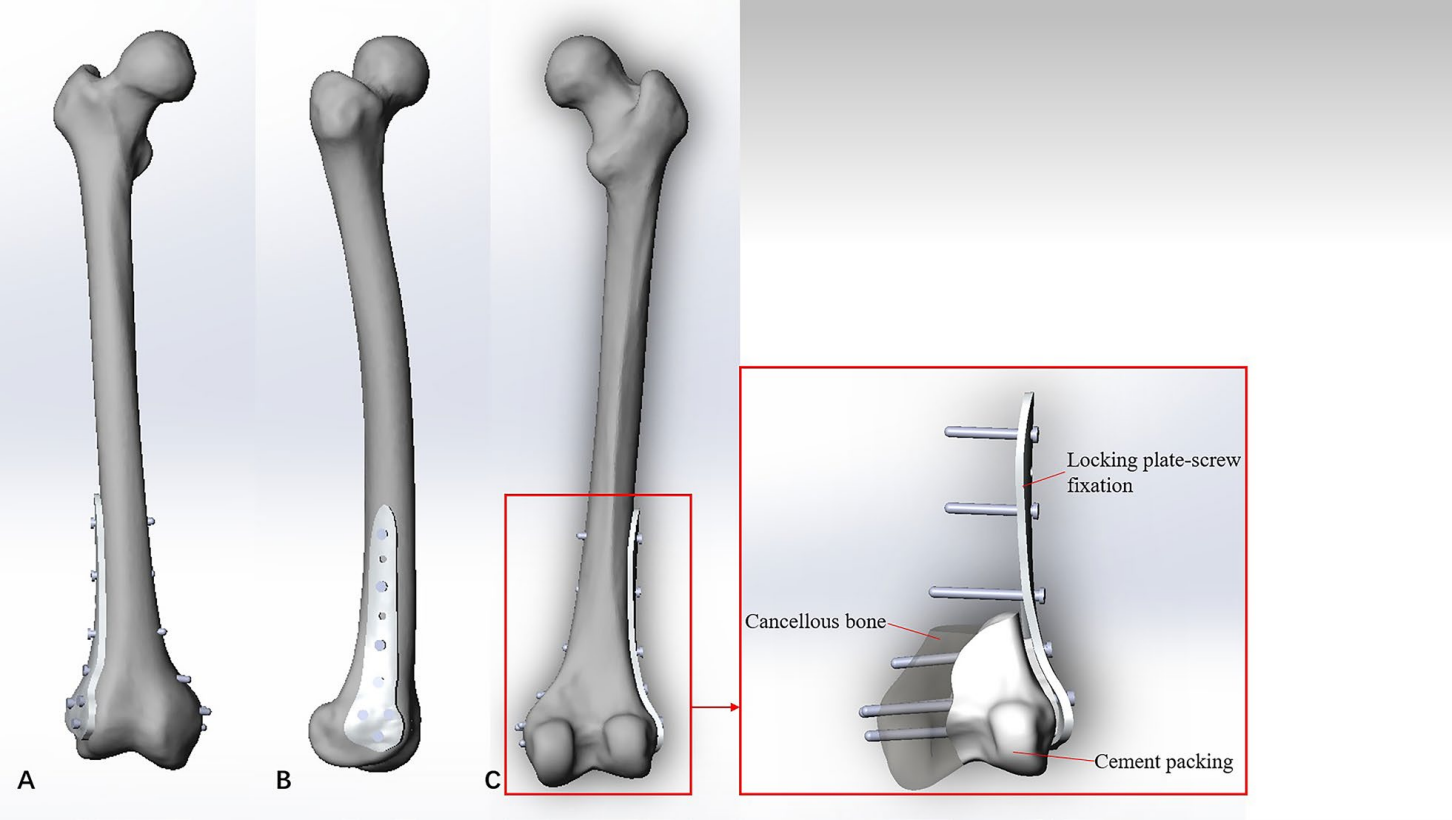
Diagram of the cement packing combined with fixation reconstruction model: **A** Front view, **B** Side view, and **C** Back view

Model #3 was created based on Model #2, and it required several components, including the distal femur locking plate, the screws, and the bone cement. The distal femur locking plate and the screws were created in Solidworks 2016 on guidance of the manufacturers’ specifications. The cavity bone defects after extensive curettage were filled with bone cement, and thus the residual cavity in Model #2 was used to replace the bone cement by Boolean Operation. All components were assembled in Solidworks 2016 (Fig. 4).

##### Model #4: cavity bone defects reconstructed by 3D-printed strut-type prosthesis combined with subchondral bone grafting

**Fig. 5:**
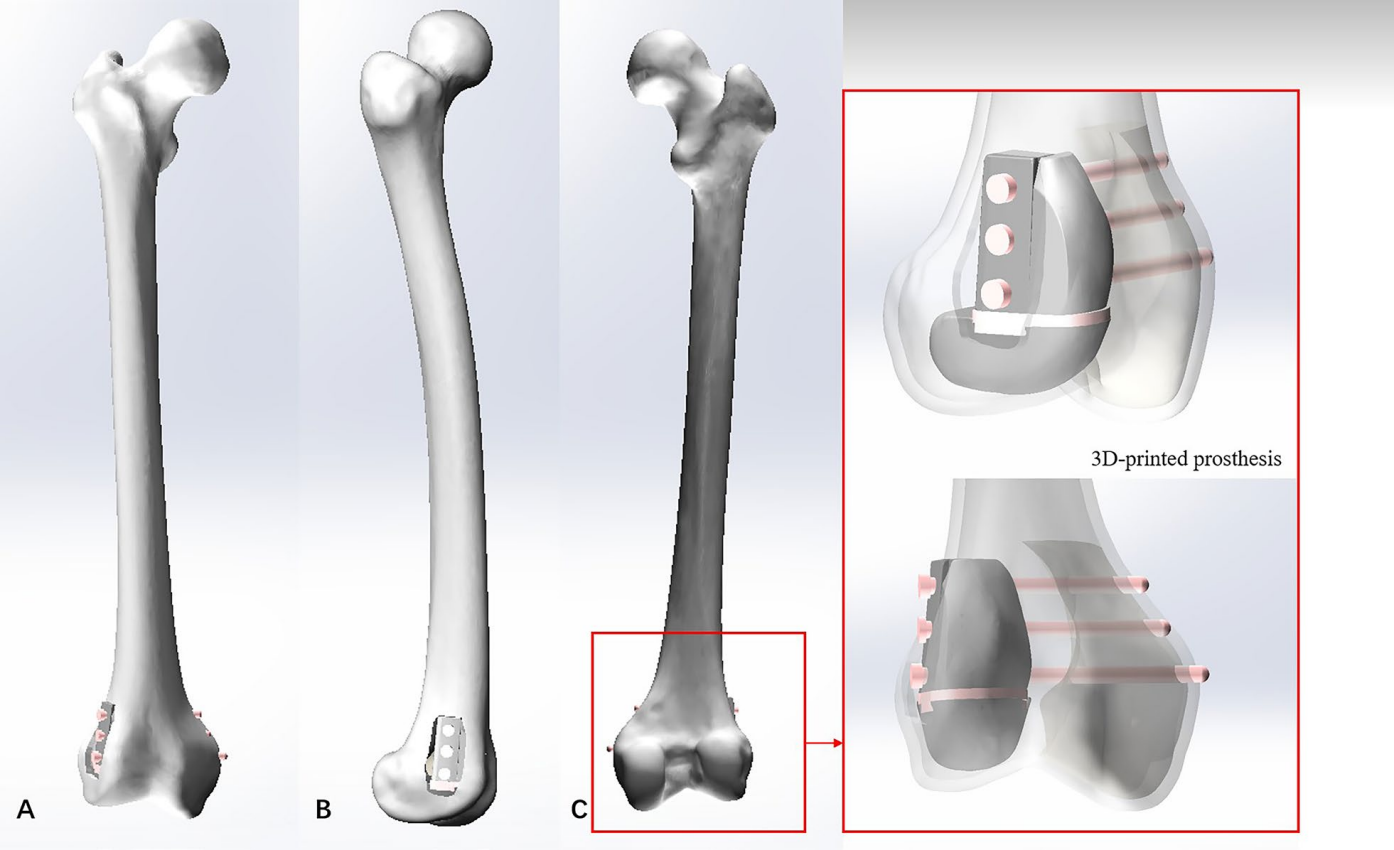
Diagram of the 3D-printed strut-type prosthetic reconstruction model: **A** Front view, **B** Side view, and **C** Back view

Model #4 was assembled with the 3D-printed porous strut-type prosthesis and Model #2 (Fig. 5). The 3D-printed porous strut-type prosthesis was created by the design data from our previous study: The size of the trapezoid-shaped strut was 12 mm × 28 mm × 38 mm (max-length × max-width × max-height), the size of the turtle shell-shaped strut A was 13 mm × 28 mm × 37 mm (max-length × max-width × max-height), and the size of the turtle shell-shaped strut B was 48 mm × 32 mm × 20 mm (max-length × max-width × max-height). A pore size of 500 μm and 70% porosity was used to simulate the trabecular bone according to data from Torres-Sanchez’s study, which confirmed that these parameters could improve osseointegration [17].

#### Material assignment and mesh

All materials were set as homogeneous, isotopic, and elastic linearly properties, and the material properties were assigned, respectively, in Ansys 2019 R3 (ANSYS, Inc. Pennsylvania, USA). The elastic modulus and Poisson’s ratio of these materials are listed in Table 2, according to previous study [18]. Furthermore, all materials of FE models were meshed individually, and the element size was 1.0 mm. The number of elements (C3D10) for each model were 2545815 (Model #1), 501438 (Model #3), and 1738013 (Model #4), respectively.

**Table 2:** Material properties of the bone and implants

Materials	Modulus of elasticity (MPa)	Poisson’s ratio
Cortical bone	13,700	0.30
Cancellous bone	1850	0.30
Bone cement	2070	0.35
Solid titanium (Ti6Al4V)	110,000	0.30
Porous titanium (Ti6Al4V)	1500	0.30

#### Loads and constraints

**Fig. 6:**
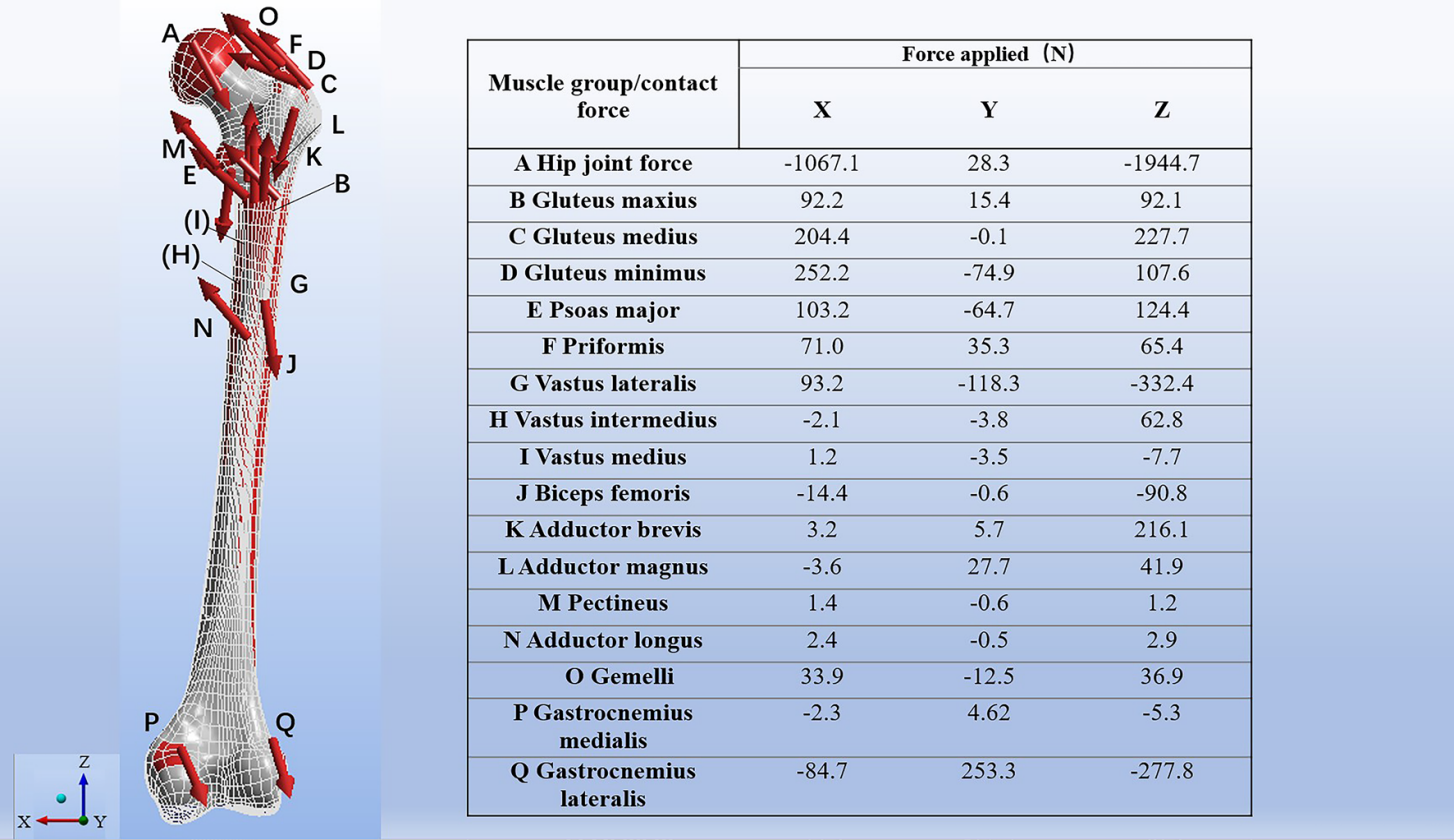
The hip joint-femur muscle multiple force was applied to these femur models, and the distal condyle articular surface was fixed

A physiological loading representing an instant at forty five percent of the gait cycle (the second part of the single-leg support period) was selected in Ansys 2019 R3. The femoral insertion areas of muscles were mapped on to the surface of each model according to a study by Viceconti et al. [19], and the hip joint-femur muscle multiple force was loaded on each model as recommended by Taylor et al. [20]. The articular surface of distal condyle was restricted in all directions. (Fig. 6).

In this study, the contact of the cortical bone–trabecular bone interface was assigned as bonded in Ansys 2019 R3. For Model #3, a frictional coefficient of 0.3 was used for contact boundary with finite sliding between the bone and the distal femur locking plate, as well as the screws. In order to simplify the experiment, it is assumed that the bone graft had united with the host bone. However, since the cement–graft bone interface cannot achieve osseointegration, the contacts of this interface were assigned as frictional with the friction coefficient of 0.3. For Model #4, the contact of the porous implant–bone interfaces was all set as bonded to simulate the mechanical effects of the osseointegration.

#### Finite element analysis

All FE models were input to Ansys 2019 R3, and the algorithm was set as Patch Conforming. The results of FEA reveled the biomechanical performance regarding two major features: the displacement and the stress.

## Results

### The results of clinical follow-up

**Fig. 7:**
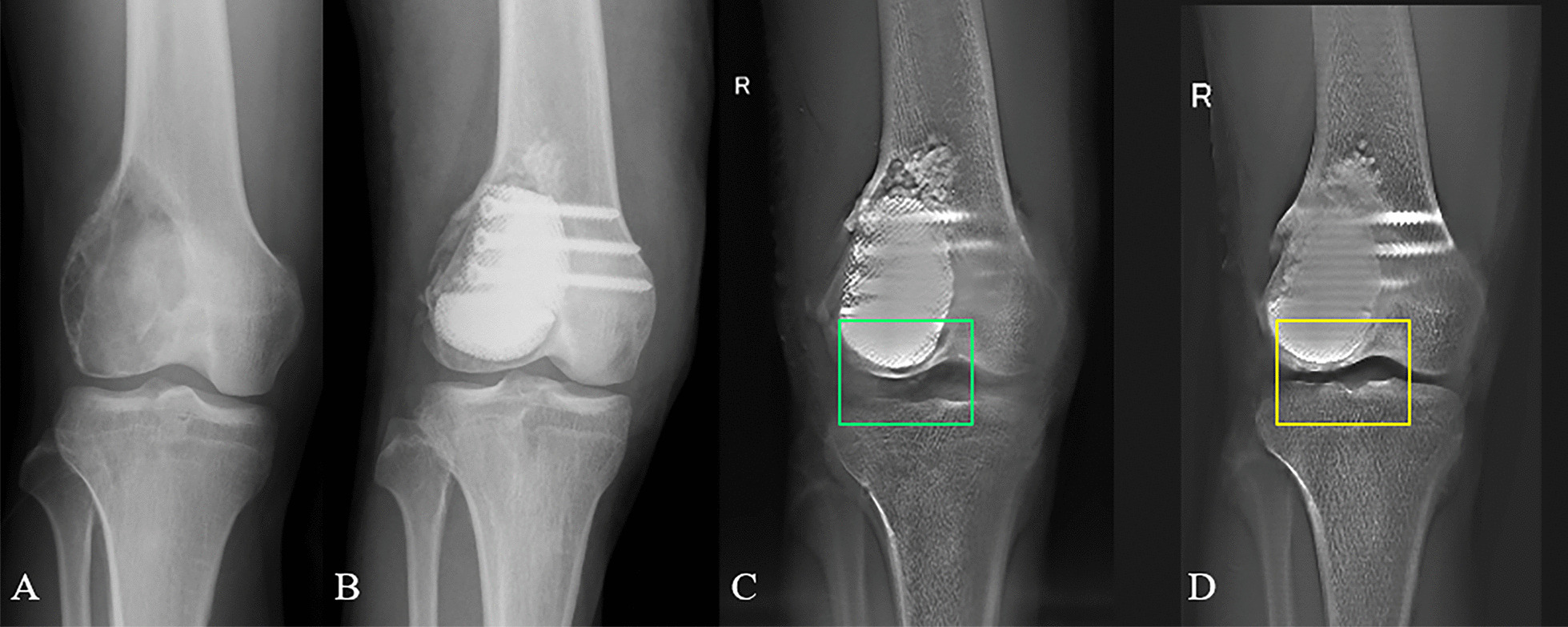
Postoperative T-SMART showed osseointegration: **A** a AP view of a 29 years olf male patient with GCTB. **B** Extended curettage, subchondral bone grafting and 3D-printed strut-type prosthetic reconstruction were performed. **C** T-SMART in postoperative day 1 showed interfacial gap between bone and implant (green box). **D** T-SMART taken at 2 years after surgery showed that excellent osseointegration

**Fig. 8:**
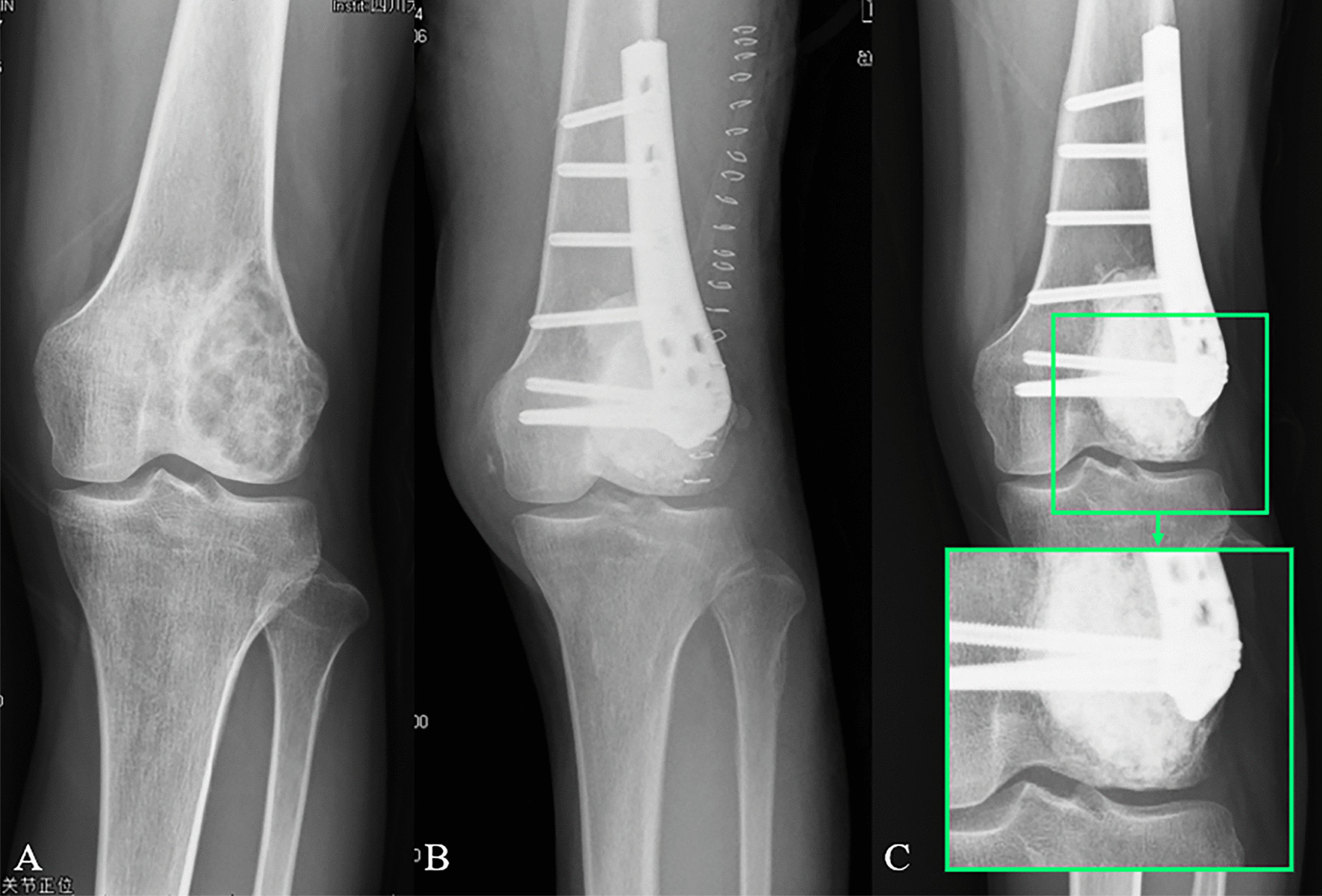
Preoperative and postoperative X-ray evaluations: **A** a AP view of a 29 years old male patient with GCTB. **B** Extended curettage, cement packing, subchondral bone grafting and plate-screws fixation were performed. **C** A sclerotic rim occurred (green box), and an interfacial gap between bone and cement can be observed

None of the patients exhibited tumor local recurrence or distant metastasis, and all patients were alive at the last follow-up. Compared to the cement group, the 3D-printing group had a slightly higher MTST score (28.4 ± 1.8 vs 26.2 ± 2.8, *P* = 0.176), and a better knee motion (range 0°–143.8° ± 6.0° vs 0°–132.6° ± 11.7°, *P* = 0.093). Although those differences were not statistically significant, definite advantages in terms of osteointegration and protection of subchondral bone was observed in the 3D-printing group. All 5 patients from 3D-printing group achieved bone graft fusion, and the absence of interfacial gap between bone and implant can be observed in T-SMART, which implied that good osseointegration was formed (Fig. 7). By contrast, since the cement–bone/graft interface cannot achieve bony ingrowth, no evidence of osteointegration could be found in the cement group. Interfacial gap and even sclerotic rim can be observed in these patients on X-rays (Fig. 8).

### The results of finite element analysis

#### The displacement and stress of the normal femur

**Fig. 9:**
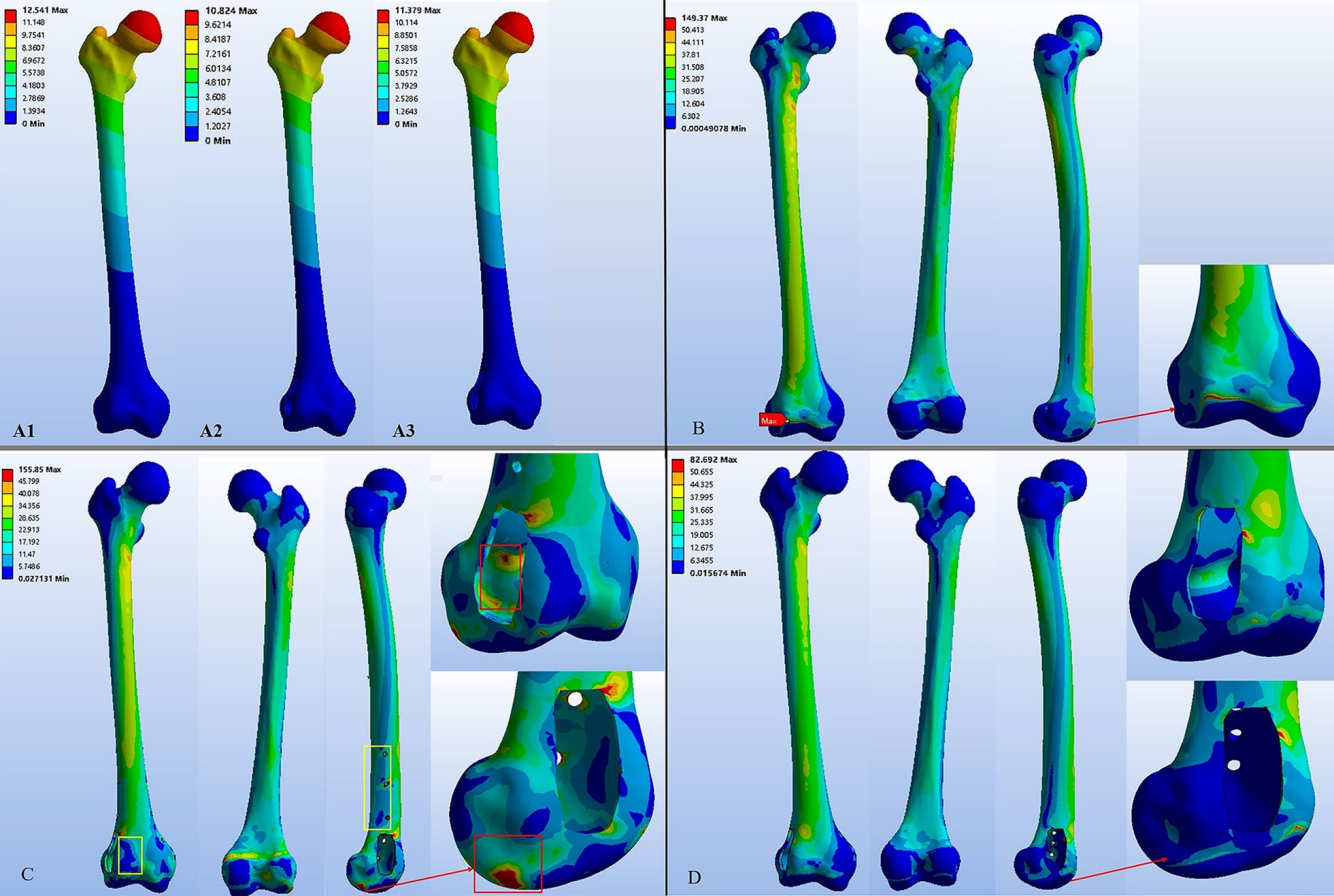
The displacement and stress distribution of femurs: **A1**–**3** The displacement of femur in Model #1, #3, and #4. **B** The stress distribution of the normal femur. **C** The stress distribution of femur in Model #3, stress shielding (yellow box) and stress concentration (red box) occurred. **D** The stress distribution of femur in Model #4

The peak displacement (12.54 mm) occurred at the center of femoral head and the top of great trochanter, and the displacement gradually decreased from the proximal to distal femur along the femoral shaft (Fig. 9A1). The stress distribution of the normal femur is observed in Fig. 9B. The hip joint force transmitted from the femoral neck to the femoral condyles with stress evenly distributed through the whole femur, and relatively high stress concentration occurred at the lesser trochanter (48.80 Mpa) and the anterior distal femur shaft (36.26 Mpa). In general, the FE results of the normal femur were in line with those of previous studies [21, 22].

#### The displacement and stress of the femur repaired with cement packing combined with extra fixation

Compared to the normal femur, similar patterns of displacement were observed in the femur of Model #3. The displacement value in the lateral femoral condyle was ranged from 0.01 to 0.06 mm≈0.01 to 0.04 mm (Model #1), and the peak displacement was 10.43 < 12.54 mm (Model #1), suggesting that cement packing combined with subchondral bone grafting and extra fixation could achieve stable fixation of the cavity bone defects (Fig. 9A2). However, this reconstruction method changed the transmission mode of the femoral mechanics, and there was a significant difference in stress distribution between Model #3 and #1. Stress shielding was located at the distal femoral shaft just beneath the plate, and the Von Mises stress in this region ranged from 1.96 to 8.28 Mpa which was lower than that of the normal femur (6.04–14.56 Mpa). Lower Von Mises stress (3.22–6.50 Mpa) were also observed in the anterior side of the lateral femoral condyle compared with that of the normal femur (12.54–22.56 Mpa). Furthermore, high stress concentration (84.45 Mpa) occurred in the posterior–inferior side of the lateral femoral condyle facing the articular surface of lateral tibiofemoral joint (Fig. 9C).

#### The displacement and stress of the femur repaired with 3D-printed strut-type prosthesis

The displacement distribution of the femur repaired by 3D-printed strut-type prosthesis was close to the normal femur (Fig. 9A3), and the displacement value in the lateral femoral condyle varied from 0.01 to 0.07 mm≈0.01 to 0.04 mm (Model #1). Like Model #3, the result suggested that the 3D-printed strut-type prosthesis could provide enough mechanical support for the femur with cavity bone defect. The stress distribution in the femur of Model #4 was also close to the normal femur. Stress distribution tended to be continuous and homogeneous, and the peak stress was located at the site where the trapezoid-shaped strut was connected to the femoral cortex, which was lower than the yield stress of cortical bone (Fig. 9D).

#### The displacement and stress of the cement-plate-screw fixation system

**Fig. 10:**
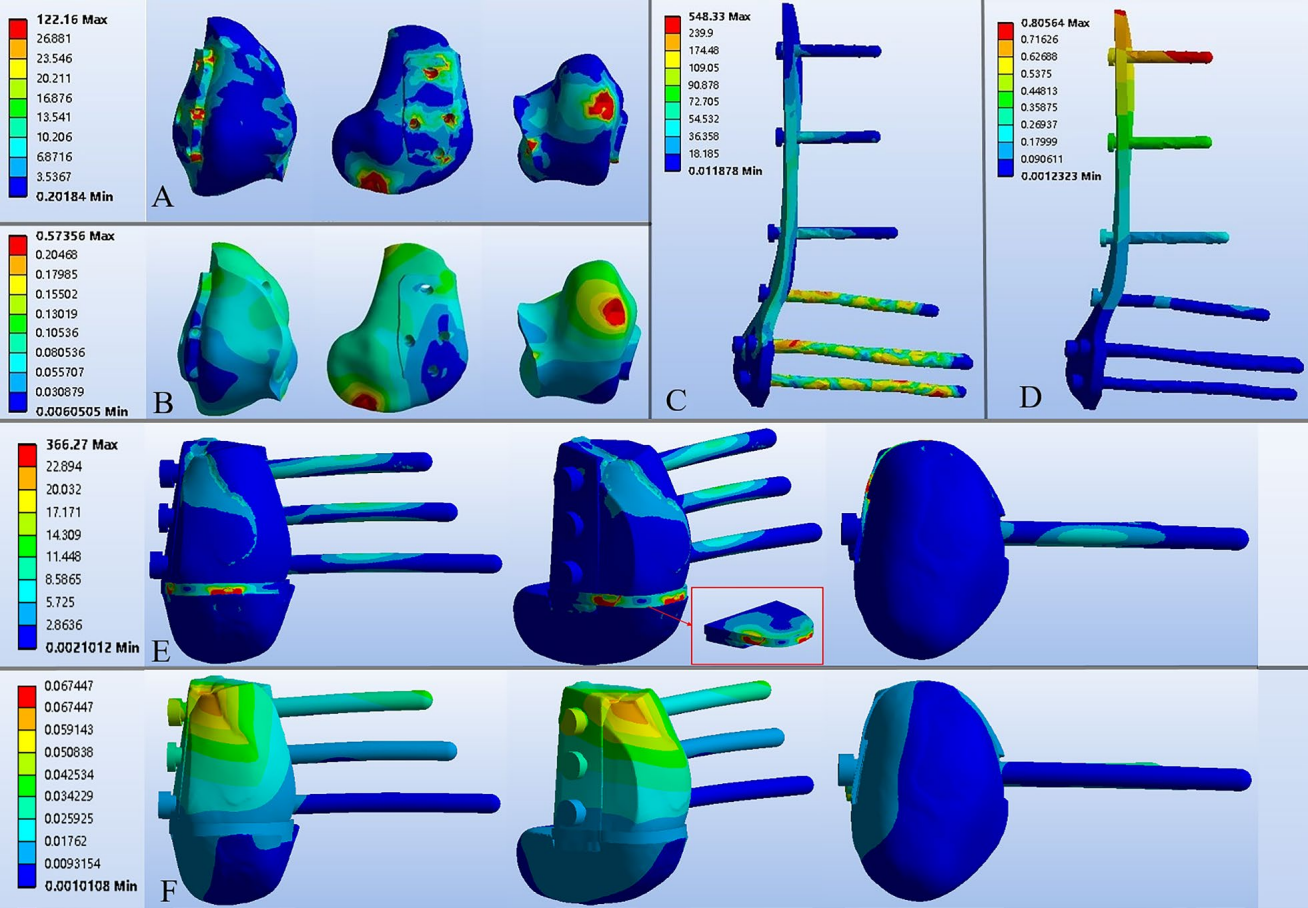
The displacement and stress distribution of implants: **A**, **B** The stress and displacement distribution of the bone cement, high stress concentration and displacement occurred at the bottom of the cement near the articular surface. **C**, **D** The stress and displacement distribution of the locking plate and screws. **E**, **F** The stress and displacement distribution of the 3D-printed strut-type prosthesis

In Model #3, the direction of displacement was vertically downward, with peak displacement concentrated on the top of the implant and decreasing progressively and distally (Fig. 10D). The peak displacement of Model #3 was 0.81 mm, which was significantly higher than that of Model #4 (0.07 mm). The contact stress distributed equally around the locking plate, except the regions where it was contacted with the screws, and the peak value (548.33 Mpa) appears at the top screw hole. Interestingly, the peak displacement (0.57 mm) and the peak stress (122.16 Mpa) of the bone cement were located at its posterior–inferior side contacting with the stress concentration area of the femoral condyle mentioned in Fig. 9C.

#### The displacement and stress of the 3D-printed strut-type prosthesis

For the 3D-printed strut-type prosthesis, the stress distributed mainly on the solid structure was located at the bottom of the trapezoid-shaped strut. In contrast, less stress was transmitted to the turtle shell-shaped struts made of porous titanium. Meanwhile, the turtle shell-shaped struts had a smaller displacement compared with the cement-plate-screw system. In addition, the screws fixed at trapezoid-shaped strut can be divided into three regions: the lateral, the middle, and the medial region. Significant displacement and local stress concentration has been found on the middle region of the screws and the top of the trapezoid-shaped strut (Fig. 10E, F).

## Discussion

### Traditional reconstruction method faces challenge of protecting articular cartilage and subchondral bone

Surgical intervention is usually inevitable for the treatment of GCTBs due to their unique biological features, including local aggressiveness, high risk of recurrence, and easily affecting the knee joint of young adults (20–40 years) [4]. Extended intralesional curettage followed by cement packing combined with subchondral bone grafting and plate-screw fixing has been accepted as a main-stream therapy for Campanacci grade I and II GCTBs around the knee. However, several disadvantages of this reconstruction method still exist. Clinically, the exothermic reaction developed during cement hardening can cause thermal necrosis of the surrounding graft bone and the articular cartilage [4], which is associated with poor bone graft incorporation. In some cases, bone cement can even accidently leak into the graft bone making the bone healing more difficult. Another drawback for autogenous bone grafting is that the bulk grafts can hardly match the shape of the articular surface, although at the expense of increasing donor site morbidity.

Most importantly, as a non-biological reconstructive material, bone cement failed to grow biologically into the autogenous bone, which cannot achieve effective osteointegration. In our study, this condition was simulated by assigning the contacts of cement–graft bone interface as frictional with the friction coefficient of 0.3. It should be noted that to simplify the experiment and to reduce the variables, we assumed that the bone graft had united with the host bone right beneath it. However, even under the ideal conditions, high displacement and stress concentration still could be found in the contact area between the bone and the cement, especially in the posterior–inferior side of the lateral femoral condyle facing the articular surface of lateral tibiofemoral joint. High displacement indicated that a poor stability of the surgical reconstruction and inappropriate stress concentration increased the possibility of pathological fracture and implant reversion [23]. The micromotion and stress concentration may affect the cement mechanical behavior. Consequently, a sclerotic rim occurred, which separate the cement from the surrounding bone [24]. Some researchers believed that this rim decreased the shock-absorbing ability of the subchondral bone, and the articular subchondral bone could be damaged by the fretting wear due to the separation around the cement [25]. The subchondral bone, an effective shock absorber, plays a vital role in maintaining the shape and the stability of the knee joint [26]. Previous studies have stated that a subchondral bone damage could lead to postoperative mechanical failure and worse knee joint function [27, 28]. In a retrospective study by Teng et al. [28], GCTBs patients undergone extensive knee curettage followed by cement packing were classified into different groups according to the extent and depth of the subchondral bone damage, and those from the mild injury group had a lower risk of mechanical failure. In summary, our study confirmed that cement packing combined with subchondral grafting lacks protection of the articular surface and subchondral bone from a mechanical perspective.

### 3D-printed strut-type prosthesis improved biomechanical performance and achieved integrated reconstruction

The FE results of the present study suggested that even though both surgical methods could offer good initial stability and mechanical support for the femur with cavity bone defect after extend curettage, the 3D-printed strut-type prosthesis showed better biomechanical performance in both displacement and stress distribution. Compared to the conventional plate-screw fixation system, the 3D-printed strut-type prosthetic reconstruction provided a near-normal stress distribution in the representative daily activity, the second part of the single-leg support period, in the distal femur after extend curettage. There was no significant displacement found in the bone defect area, and the maximum displacement of the 3D-printed porous strut-type prosthesis was 0.07 mm, which was significantly lower than that of the cement-plate-screw systems (0.81 mm). It implied that the 3D-printed porous strut-type prosthesis provided better stability.

Some advantages of the 3D-printed strut-type prosthetic reconstruction, in contrast, may include the following. Firstly, the 3D-printed strut-type prosthesis was customized depending on the results of preoperative imaging, and it could match well with the massive-cavity bone defect as an intra-femur implantation. Thanks to its precise shape matching, less amount of bone graft was required to achieve satisfactory subchondral bone grafting. In comparison, the plate-screw fixation in Model #3 increased the extra contact area between the femur and the implants, which had changed the way of stress transmission, and the traditional reconstruction method generally suffers from the issue that the source of autogenous bone in bone grafting is limited. Secondly, the 3D-printed strut-type prosthesis with porous scaffold had outstanding advantage of osteointegration capacity. Theoretically, after the 3D-printed strut type prosthesis implanting into the cavity bone defect, it can form a tight permanent fixation between the bone–prosthesis interface in the long term. The porous scaffold has been proved to be useful in promoting the osteointegration of bone–prosthesis interface [29]. The porous trabecular-like structure with specific size and porosity not only allow ingrowth of bone tissue into the pores, but also provide a similar elastic modulus as the host bone [29, 30]. With the development of additive manufacturing technology, the porous scaffold was widely applied in the design and manufacture of titanium-based prosthesis which continues to be used in clinical practice to repair bone defects. Torres-Sanchez’s study has identified that a pore size of 500 μm and 70% porosity can be used to simulate the trabecular bone [17]. Therefore, the 3D-printed strut-type prosthesis with porous scaffold was designed with these parameters to improve the osseointegration ability of the implant. Thirdly, the articular cartilages and subchondral bone were effectively protected due to the reasonable design of the modular system: Stress was mainly distributed on the solid structure made of solid titanium and less on the turtle shell-shaped struts made of porous titanium. This favorable biomechanical characteristic was mainly possible thanks to the integrated reconstruction based on the implant’s precise shape matching and osseointegration ability. Taken together, our study confirmed that 3D-printed strut-type prosthetic reconstruction combined with subchondral bone grafting could provide enough mechanical support and improve bone ingrowth, which had incomparable advantages in protecting the articular cartilage and subchondral bone over the conventional reconstruction method. However, further clinical studies with larger sample sizes and longer-term follow-up are needed to support these results and to evaluate its clinical application value.

### The limitation and expectation

There are some limitations of this study. Firstly, this FE analysis was performed only under single-leg support condition, and analysis under conditions such as walking and stepping up and down stairs will offer more accurate data in future experiments, replicating more realistic events. Secondly, the criterion we used to evaluate the biomechanical performance is the interfragmentary theory, which did not take into account the whole bone healing process, but only the long-term condition. In addition, we noticed a detail that the maximum value of Von mises stress in the normal femur model appears at the edge of constricted surface. This finding was unexpected and perhaps can be explained due to fast stress changing at constricted boundaries. For the experiment's precision, they should be ignored when analyzing FE data. Other FE results are consistent with previous studies, which make it a validate control group.

## Conclusion

The 3D-printed strut-type prosthesis can provide effective mechanical support and enhance osseointegration due to its precise shape matching and porous scaffold structure. Additionally, it has incomparable advantages in protecting articular cartilage and subchondral bone compared to traditional reconstruction method. Thus, we recommend 3D-printed strut-type prosthetic reconstruction combined with subchondral bone grafting as a reasonable alternative for treatment of grades I or II GCTBs in distal femur.

## Data Availability

The datasets used and analyzed during the current study are available from the corresponding author on reasonable request.

## Abbreviations

GCTB: Giant Cell Tumor of Bone
3D: Three-dimensional
FEA: Finite Element Analysis
NURBS: None-Uniform Ration Basis Spine
